# Prenatal Diagnosis of 6q Terminal Deletion Associated with Coffin–Siris Syndrome: Phenotypic Delineation and Review

**DOI:** 10.3390/genes16111365

**Published:** 2025-11-10

**Authors:** Christian Peña-Padilla, David Alejandro Martínez-Ceccopieri, Evelin Montserrat García-Hernández, Lucina Bobadilla-Morales, Jorge Román Corona-Rivera

**Affiliations:** 1 Service of Genetics, Division of Pediatrics, “Dr. Juan I. Menchaca” Civil Hospital of Guadalajara, Guadalajara 44340, Mexico; christian.pena@academicos.udg.mx; 2 Doctorate in Multidisciplinary Research in Health, University Center of Tonala, University of Guadalajara, Tonala 45425, Mexico; 3 Maternal-Fetal Medicine Unit, Division of Obstetrics and Gynecology, “Dr. Juan I. Menchaca” Civil Hospital of Guadalajara, Guadalajara 44340, Mexico; alejandro.martinez@academicos.udg.mx; 4 Research Coordination, “Fray Antonio Alcalde” Civil Hospital of Guadalajara, Guadalajara 44340, Mexico; 5 Cytogenetics Unit, “Dr. Juan I. Menchaca” Civil Hospital of Guadalajara, Guadalajara 44340, Mexico; lucinabo@gmail.com; 6 Dr. Enrique Corona-Rivera Institute of Human Genetics, Health Sciences University Center, University of Guadalajara, Guadalajara 44340, Mexico

**Keywords:** *ARID1B*, fifth fingernail hypoplasia, agenesis of corpus callosum, array-CGH, prenatal diagnosis, chromosome 6q deletion, Coffin–Siris phenotype

## Abstract

Chromosome 6q deletion syndrome is a rare entity that has a highly variable clinical presentation and size of deletions. The most frequent manifestations of 6q terminal deletion are intellectual disability, facial dysmorphism, brain structural anomalies, and congenital heart defects. The phenotype is not clinically recognizable, except in those who harbor a terminal 6q deletion that includes the *ARID1B* gene, in whom features similar to Coffin–Siris syndrome (CSS) can be observed. We report the case of a female newborn with a prenatal diagnosis of a terminal deletion on 6q25.1q27, which encompasses the *ARID1B* gene, and who was diagnosed with CSS during the neonatal period. From our review, we found that facial gestalt, hypertrichosis, and fifth fingernail aplasia/hypoplasia, along with other features, such as vertebral defects and cystic hygroma (or webbed neck), correlated with the presence of a CSS causally related to 6q25.3 small deletions that include the *ARID1B* gene.

## 1. Introduction

Chromosome 6q deletion syndrome (C6qDS) is a rare entity first described by Milosevic and Kalicanin in 1975 [1] in a boy with dysmorphic features and developmental delay. Since then, several cases have been reported. The clinical recognition of C6qDS is challenging due to the variation in deletion size and genes involved. In 1997, Hopkin et al. proposed a classification of C6qDS into three groups based on the description of 60 reported cases: (A) proximal deletion (6q11-q16), (B) middle deletion (6q15-q25), and (C) terminal deletion (6q25-qter) [2]. Intellectual disability is present in all groups. Group A correlates with mild dysmorphic features and lower frequency of congenital heart defects (CHDs) and microcephaly; Group B correlates with limb anomalies and high neonatal mortality; and Group C correlates with a higher incidence of brain anomalies, but remains difficult to clinically recognize. Advances in molecular cytogenetics have broadened the clinical spectrum, identifying microdeletions and new candidate genes [3,4,5,6,7,8,9].

On the other hand, Coffin–Siris syndrome (CSS) was first described by Coffin and Siris in 1970 in three unrelated females with intellectual disability, coarse facial features, and absence of the fifth fingernail [10]. Not until 2012 did Santen et al. identify pathogenic variants in the *ARID1B* gene as the etiology of CSS type 1 (CSS1) [11], and in the same year, Tsurusaki et al. reported other CSS genes related to the SWI/SNF complex [12]. The *ARID1B* gene (6q25.3) was proposed as a candidate gene for cerebral dysgenesis in terminal 6q deletion syndrome by Backx et al. 2011 [13] and later by Michelson et al. 2012 in a boy with an interstitial deletion at 6q25, including only the genes *ARID1B* and *ZDHHC* [14]. Most patients with CSS do not have prenatal US anomalies, and those reported are mainly congenital diaphragmatic hernia (CDH), congenital heart defects (CHDs), and intrauterine growth restriction (IUGR). However, in CSS related to *ARID1B*, the most frequent anomalies detected by prenatal US are brain anomalies, including corpus callosum agenesis (CCA) [15]. Here, we report a case of a female newborn with a prenatally detected 6q terminal deletion, which encompasses the *ARID1B* gene, in whom classic CSS was clinically diagnosed at birth. Additionally, we review all previous reports with similar deletions on chromosome 6q.25.3.

## 2. Materials and Methods

Written informed consent was obtained from the parents for publication. Amniotic fluid was collected via transabdominal amniocentesis. Fetal cells were cultured in situ and prepared for G-banded karyotyping. Chromosomal analysis was performed on metaphase spreads, with 20 cells counted. The karyotype was interpreted and reported according to ISCN, 2020. Genomic DNA was extracted from peripheral blood leukocytes using a commercial extraction kit, according to the manufacturer’s protocol. aCGH was performed using the commercial oligonucleotide microarray platform KaryoNIM^®^60K. Data were analyzed using Agilent Cytogenomics Agilent Technologies, Inc., Santa Clara, California, USA Software v5.0.2.5 [16]. Detected copy number variations (CNVs) were filtered and classified following the guidelines of ACMG.

## 3. Case Report

**Figure 1:**
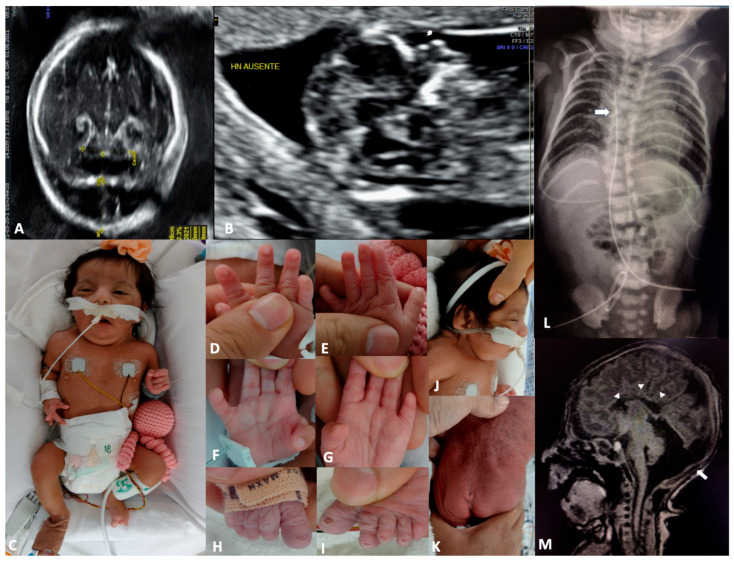
Clinical features in the *proposita*. (**A**,**B**) Prenatal ultrasound showing a cystic hygroma, mega cisterna magna, and absent nasal bone. (**C**) Coarse facial features. (**D**–**I**), Nail aplasia/hypoplasia, short fifth fingers. (**J**) Dysplastic, low-set ears, and redundant nuchal skin. (**K**) Deep sacral skin dimple. (**L**) Thorax radiography showing mild scoliosis and T6 hemivertebra (arrow). (**M**) Sagittal view of a brain MRI showing agenesis of the corpus callosum (arrowheads), severe hypoplasia of the cerebellum, and pseudocystic dilatation of the posterior fossa (arrow).

**Figure 2:**
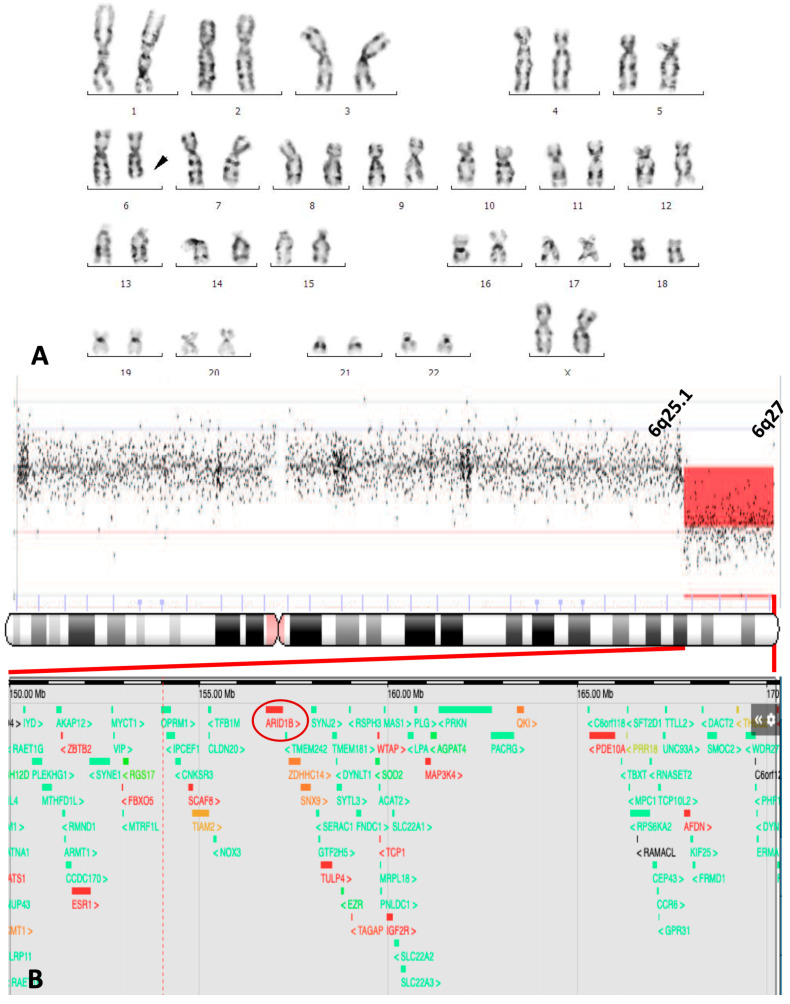
Cytogenetic findings. (**A**) Karyogram (G-banding) of amniocytes demonstrating the female complement with a deletion on the long arm of chromosome 6:46, XX, del(6)(q23) (arrow). (**B**) aCGH Profile of chromosome 6 with a detailed view for the bands 6q25.1 to 6q27 and genes involved, including *ARID1B* (red circle).

The *proposita* was the first child of a young, healthy, and non-consanguineous couple. No family history of miscarriages or malformations was reported. During the first trimester, a fetal ultrasound detected a cystic hygroma (CH) and absence of the nasal bone (Figure 1A,B). At 18 weeks of gestation, amniocentesis and karyotyping were performed, reporting 46, XX, del(6)(q23) (Figure 2A). Despite prenatal diagnosis, the parents decided to continue the pregnancy. She was born at the 34th week of gestation by cesarean section. The Apgar score was 7–8 at 3 and 5 min, respectively. At birth, weight was 1840 g (P16), height was 41 cm (P10), and OFC was 32 cm (P76). Physical examination revealed coarse facial features with generalized hypertrichosis, dysplastic ears, a short neck with redundant nuchal skin, a broad thorax with widely spaced nipples, short hands with brachydactyly, nail hypoplasia of fingers 1–4, and nail aplasia of the left fifth finger. The feet showed nail aplasia of the fifth toes. A deep sacral skin dimple was also noted (Figure 1C–K). Thorax radiography revealed a hemivertebra in T6 (Figure 1L). Brain MRI showed agenesis of the corpus callosum, cerebellar hypoplasia, and pseudocystic dilatation of the posterior fossa (Figure 1M). Echocardiogram showed an ostium secundum atrial septal defect (ASD), along with a ventricular septal defect (VSD). Renal ultrasonography was normal. A CGH array was performed, revealing arr [16] 6q25.1q27 (150321670_170537245)x1 (Figure 2B). The deletion spanned 20.2 megabases, involving at least 74 genes, spanning morbid genes as *SYNE1*, *ARID1B*, *PDE10A*, *DLL1*, and *TBP* (Figure 2B, lower panel in the enlargement). A normal karyotype was obtained for both parents. The *proposita* died at three months of age due to sepsis and multiorgan failure; an autopsy was not performed.

## 4. Discussion

Pure chromosome 6q terminal deletions are relatively rare, with a frequency of about 0.05% in patients with intellectual disability and multiple malformations [9]. Although deletions across all chromosome 6q have been described, most recent research has focused on subtelomeric deletions in the cytoband 6q27, adding a pure subtelomeric deletion subgroup for the original Group C proposed by Hopkin et al. [2]. Regarding this subgroup, some candidate genes (*DLL1*, *THBS2*, *PHF10*, and *C6orf70*) have been proposed to explain the observed brain anomalies [4,5,6,8,9,17]. Expanding this subject, in 2005, Eash et al. described a correlation between deletion spans 6q26-q27 with congenital heart defects, genital hypoplasia, a short neck, and retinal anomalies, and pure subtelomeric deletions with dysmorphic features and brain anomalies, including ventriculomegaly/hydrocephaly [18]. Our patient, along with the 14 other cases summarized in Table 1, was analyzed to establish possible specific genotype–phenotype correlations, as detailed below.

A key objective of our analysis was to correlate specific genetic loci within Group C with distinct phenotypes. We noted that *ARID1B* gene haploinsufficiency (proximal at Group C, 6q25.1) correlates with the distinctive nail hypoplasia/aplasia of the fifth finger. This recognizable clinical manifestation is observed in 68% of patients with CSS1 [19]. Our patient is one of only three terminal 6q deletion cases to date that exhibit a recognizable CSS phenotype (along with Stevens et al. [20] and Meng et al. [21]). This finding suggests that the CSS phenotype in other 6q terminal deletion cases may be masked by haploinsufficiency of other contiguous subtelomeric genes. This is supported by the fact that only patients with small interstitial deletions, including *ARID1B*, display a more evident CSS phenotype [14,19,22]. Other seemingly specific features were hypertrichosis and corpus callosum agenesis (Table 1). Our case is particularly significant due to the presence of cystic hygroma (CH), a feature previously reported only in a few patients harboring deletions between 6q23 and 6q27 [23,24,25]. Also, Meng et al. reported a case with a webbed neck and karyotype 46, XX, del(6)(q25) [21], suggesting that CH is a clinical feature in terminal deletion but not in subtelomeric deletion only. No genes in this chromosomal region are known to be involved in CH or lymphatic system development. All five patients had CHD, specifically AVD and VSD, except for those reported by Shen-Shwartz et al. and Meng et al. [21,23], in which an atrioventricular septal defect was reported, leading to the suspicion of a relationship between CHD and CH in C6qDS patients.

**Figure 3:**
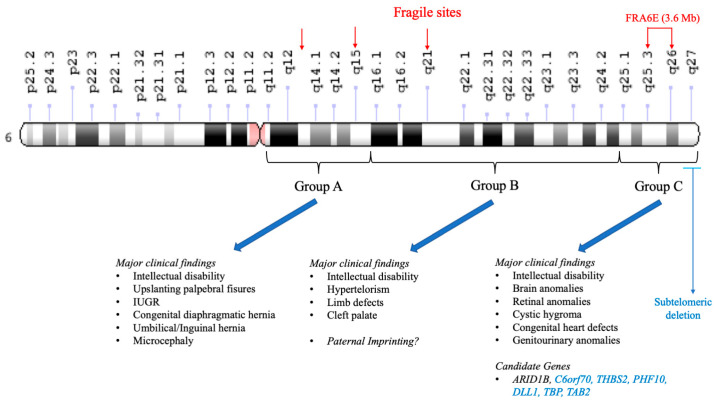
Chromosome 6 deletion groups: 6q deletion Groups A, B, and C proposed by Hopkin et al. (1997) [2]. Major clinical findings of each group are noted. Known fragile sites at q13, q21, and q26 are shown (red arrows). Subtelomeric group (blue arrow). Candidate genes in the Group C subtelomeric region are shown in blue. FRA6E spans from 6q25.3 to 6q26. IUGR: Intrauterine growth restriction.

The clinical variability among case reports could be explained by the wide range of deletion sizes. Chromosome 6q contains several known fragile sites (q13, q21, and q26) [26], in addition to a preferential breakpoint at 6q25 first noted by Valtat et al. in 1992 [25]. This region was later characterized as FRA6E, a fragile site spanning 3.6 Mb from 6q25.3 to 6q26 [27] (Figure 3), resulting in a highly variable deletion size and, consequently, diverse clinical outcomes.

**Table 1:** Clinical and cytogenetic features in patients with distal pure 6q deletion syndrome (Group C).

	Reference														
	[1]	[28]	[29]	[20]	[23]	[24]	[21]		[25]	[2]	[30]	[31]	[32]	[33]	Present Case
Patient				2			1	2	4	3	5	4	4		
Sex	M	M	M	M	M	F	M	F	M	M	F	F	M	F	F
Age at last examination	2 yr	9 mo	3 yr	1 yr	21 wk	Fetus	4 mo	2 yr	10 yr	2 yr	37 yr	9 yr	10 mo	18 mo	3 mo
***Prenatal findings***															
Intrauterine growth retardation	−	−	−	−	+	−	−	−	−	−	NS	−	−	+	−
Cystic hygroma	−	−	−	−	+	+	−	−	−	−	NS	−	−	−	+
Oligohydramnios	−	−	−	−	−	−	−	−	−	−	NS	−	−	+	−
Ventriculomegaly	−	−	−	−	+	+	−	−	−	−	NS	−	+	−	−
Hydrops fetalis	−	−	−	−	+	−	−	−	−	−	NS	−	−	−	−
Hydrothorax	−	−	−	−	−	−	−	−	−	−	NS	−	−	−	−
Absent nasal bone	−	−	−	−	−	−	−	−	−	−	NS	−	−	−	+
Diaphragmatic hernia	−	−	−	−	+	−	−	−	−	−	NS	−	−	−	−
***Natal/postnatal findings***															
Birth length cm (percentile)	NS	NS	51 (P50)	48 (P60)	23 (P25)	NS	44	NS	NS	55.5 (P5)	NS	49 (P50)	47 (P1)	48	41 (P10)
Birth weight g (percentile)	NS	2670 (P1)	3600 (P54)	2870 (P60)	280 (P10)	NS	2220	1800	NS	2600 (P10)	NS	2800 (P25)	2800 (P3)	2000	1840 (P16)
OFC at birth cm (percentile)	NS	NS	35.5 (P50)	32 (<P3)	16 (P25)	NS	30.8	NS	NS	44 (>P95)	NS	34 (P50)	31 (<P1)	30	32 (P76)
Developmental delay/intellectual disability	+	+	+	+	NA	NA	+	+	+	+	+	+	+	+	+
Microcephaly	Yes	+	+	+	−	NS	+	−	−	−	NS	−	+	+	−
Sparse scalp hair	−	+	−	−	NS	−	+	−	−	+	−	−	−	−	−
Thick eyebrows	−	−	−	−	NS	−	−	−	−	−	+	−	+	−	+
Long eyelashes	−	−	−	−	NS	−	−	−	−	−	NS	−	+	−	+
Dacryostenosis	−	−	−	NS	NS	−	−	−	NS	+	NS	−	−	−	−
Flat nasal bridge	−	+	+	−	+	−	+	+	−	−	−	+	−	+	+
Thick alae nasi	−	+	+	−	−	NS	−	−	−	−	+	−	−	−	+
Anteverted nose	−	+	−	+	+	+	+	−	−	+	−	−	−	+	+
Long philtrum	+	+	+	+	+	NS	+	+	−	+	−	−	+	−	+
Large mouth	+	−	+	+	−	+	−	+	+	−	+	−	−	−	+
Dysplastic ears	+	−	+	−	+	+	+	+	−	+	+	+	+	+	+
Hypertrichosis	−	−	−	+	−	−	−	+	−	−	NS	−	NS	−	+
Fifth fingernail aplasia/hypoplasia	−	−	−	+	−	NS	−	+ *	−	NS	NS	−	−	−	+
Brachydactyly	+	−	−	−	−	NS	−	NS	No	NS	NS	−	−	+	+
Hemivertebrae	NS	NS	NS	NS	−	NS	NS	NS	NS	NS	NS	−	NS	NS	+
***Congenital heart defects***															
Atrioventricular septal defect	−	−	−	−	+	−	−	+	−	+	NS	−	−	−	−
Ventricular septal defect	−	+	−	−	−	+	+	−	−	−	NS	−	−	+	+
Atrial septal defect	−	−	−	−	−	−	−	−	−	−	NS	−	+	+	+
Partially anomalous pulmonary venous drainage	−	−	+	−	−	−	−	−	−	−	NS	−	−	−	−
*Cor triatriatum*	−	−	−	−	−	−	−	+	−	−	NS	−	−	−	−
Pulmonary vein stenosis	−	−	−	−	−	−	−	−	−	+	NS	−	−	−	−
***Genitourinary anomalies***															
Hydronephrosis	+	−	−	−	−	+	−	−	−	+	−	−	−	−	−
Duplicated collecting system	−	−	−	−	+	−	−	−	−	−	−	−	−	−	−
Renal cyst	+	−	−	−	−	−	−	−	−	−	−	−	−	−	−
Cryptorchidism	+	+	−	−	−	NA	+	NA	−	−	NA	NA	−	NA	NA
Penoscrotal webbing	−	−	−	−	−	−	−	−	−	−	−	−	+	−	−
Clitoromegaly	−	−	−	−	−	−	−	−	−	−	−	−	−	+	−
Prominent labia minora	−	−	−	−	−	−	−	−	−	−	−	−	−	+	−
***Cerebral dysgenesis***															
Corpus callosum agenesis (A)/hypoplasia	NS	NS	NS	−/−	+/−	−/−	+/−	−/−	NS	−/−	−/+	−/+	+/−	+/−	+/−
Ventriculomegaly/hydrocephaly	NS	NS	NS	−/−	−/−	−/−	−/−	+/−	NS	−/+	−/−	−/−	−/−	−/−	−/−
Colpocephaly	NS	NS	NS	−	−	−	−	−	NS	−	+	+	+	−	−
Arhinencephaly	NS	NS	NS	−	+	−	−	−	NS	−	−	−	−	−	−
Cerebellar hypoplasia	NS	NS	NS	−	−	−	−	−	NS	−	−	−	−	−	+
Other anomalies	HD, PrH	CP, HD	Cd	PrH	CP, DH	DH, SUA	AA, CP, PrT, STTh	TGC, RNS	−	EPi, RPi	S	−	S	−	RNS
***Cytogenomic findings***															
Cytogenetic band deleted	6q25	6q25	6q24	6q25	6q23	6q24.3	6q24.3	6q25.3	6q25	6q25.2	6q25.3	6q25.2	6q25.3	6q24	6q25.1
*ARID1B* gene deletion	?	?	+	+	+	+	+	?	?	+	+	+	+	+	+
CSS clinically recognizable	−	−	−	+	−	−	−	+	−	−	−	−	−	−	+

M, male; F, female; IUGR, NS, non-stated; NA, not applicable, intrauterine growth restriction; CH, cystic hygroma; DD, developmental delay; PAPVD, partially anomalous pulmonary venous drainage; RVH, right ventricular hypertrophy; AVSD, atrioventricular septal defect; DCS, duplicated collecting system; CCA, corpus callosum agenesis; SUA, single umbilical artery; CCH, corpus callosum hypoplasia; ASD-OS, atrial septal defect ostium secundum; CP, cleft palate; CSS: Coffin-Siris syndrome. * seen on photograph, not mention it in text.

## 5. Conclusions

Chromosome 6q deletion syndrome is a rare chromosomal abnormality with a highly variable clinical presentation, making it very difficult to clinically recognize. The typical features of CSS, along with other anomalies, such as vertebral defects and CH (webbed neck), appear to correlate with the presence of CSS related to small deletions on 6q25.3 that include the *ARID1B* gene. Further large cohort studies are required to better understand its clinical variability and identify potential interactions between genes surrounding *ARID1B*.

## Data Availability

The original contributions presented in this study are included in this article. Further inquiries can be directed to the corresponding author.

## Abbreviations

The following abbreviations are used in this manuscript:

ASD	Atrial septal defect
CDH	Congenital diaphragmatic hernia
CH	Cystic hygroma
CHD	Congenital heart defect
CSS	Coffin–Siris syndrome
C6qDS	Chromosome 6q deletion syndrome
US	Ultrasound
VSD	Ventricular septal defect

## References

[1] Milosević J., Kalicanin P. (1975). Long arm deletion of chromosome no. 6 in a mentally retarded boy with multiple physical malformations. J. Ment. Defic. Res..

[2] Hopkin R.J., Schorry E., Bofinger M., Milatovich A., Stern H.J., Jayne C., Saal H.M. (1997). New insights into the phenotypes of 6q deletions. Am. J. Med. Genet..

[3] Caselli R., Mencarelli M., Papa F., Uliana V., Schiavone S., Strambi M., Pescucci C., Ariani F., Rossi V., Longo I. (2007). A 2.6 Mb deletion of 6q24.3-25.1 in a patient with growth failure, cardiac septal defect, thin upperlip and asymmetric dysmorphic ears. Eur. J. Med. Genet..

[4] Mosca A., Callier P., Masurel-Paulet A., Thauvin-Robinet C., Marle N., Nouchy M., Huet F., Dipanda D., De Paepe A., Coucke P. (2010). Cytogenetic and array-CGH characterization of a 6q27 deletion in a patient with developmental delay and features of Ehlers-Danlos syndrome. Am. J. Med. Genet. A.

[5] Gerber J.C., Neuhann T.M., Tyshchenko N., Smitka M., Hackmann K. (2011). Expanding the clinical and neuroradiological phenotype of 6q27 microdeletion: Olfactory bulb aplasia and anosmia. Am. J. Med. Genet. A.

[6] Rigon C., Salviati L., Mandarano R., Donà M., Clementi M. (2011). 6q27 subtelomeric deletions: Is there a specific phenotype?. Am. J. Med. Genet. A.

[7] Nowaczyk M.J., Carter M.T., Xu J., Huggins M., Raca G., Das S., Martin C.L., Schwartz S., Rosenfield R., Waggoner D.J. (2008). Paternal deletion 6q24.3: A new congenital anomaly syndrome associated with intrauterine growth failure, early developmental delay and characteristic facial appearance. Am. J. Med. Genet. A.

[8] Peddibhotla S., Nagamani S.C.S., Erez A., Hunter J.V., Holder J.L., Carlin M.E., Bader P.I., Perras H.M.F., Allanson J.E., Newman L. (2015). Delineation of candidate genes responsible for structural brain abnormalities in patients with terminal deletions of chromosome 6q27. Eur. J. Hum. Genet..

[9] Thakur M., Bronshtein E., Hankerd M., Adekola H., Puder K., Gonik B., Ebrahim S. (2018). Genomic detection of a familial 382 Kb 6q27 deletion in a fetus with isolated severe ventriculomegaly and her affected mother. Am. J. Med. Genet. A.

[10] Coffin G.S., Siris E. (1970). Mental retardation with absent fifth fingernail and terminal phalanx. Am. J. Dis. Child..

[11] Santen G.W., Aten E., Sun Y., Almomani R., Gilissen C., Nielsen M., Kant S.G., Snoeck I.N., Peeters E.A.J., Hilhorst-Hofstee Y. (2012). Mutations in SWI/SNF chromatin remodeling complex gene ARID1B cause Coffin-Siris syndrome. Nat. Genet..

[12] Tsurusaki Y., Okamoto N., Ohashi H., Kosho T., Imai Y., Hibi-Ko Y., Kaname T., Naritomi K., Kawame H., Wakui K. (2012). Mutations affecting components of the SWI/SNF complex cause Coffin-Siris syndrome. Nat. Genet..

[13] Backx L., Seuntjens E., Devriendt K., Vermeesch J., Van Esch H. (2011). A balanced translocation t(6;14)(q25.3;q13.2) leading to reciprocal fusion transcripts in a patient with intellectual disability and agenesis of corpus callosum. Cytogenet. Genome Res..

[14] Michelson M., Ben-Sasson A., Vinkler C., Leshinsky-Silver E., Netzer I., Frumkin A., Kivity S., Lerman-Sagie T., Lev D. (2012). Delineation of the interstitial 6q25 microdeletion syndrome: Refinement of the critical causative region. Am. J. Med. Genet. A.

[15] van der Sluijs P.J., Joosten M., Alby C., Attié-Bitach T., Gilmore K., Dubourg C., Fradin M., Wang T., Kurtz-Nelson E.C., Ahlers K.P. (2023). Discovering a new part of the phenotypic spectrum of Coffin-Siris syndrome in a fetal cohort. Genet. Med..

[16] McGowan-Jordan J., Hastings R.J., Moore S. (2020). ISCN 2020: An International System for Human Cytogenomic Nomenclature (2020).

[17] Wadt K., Jensen L.N., Bjerglund L., Lundstrøm M., Kirchhoff M., Kjaergaard S. (2012). Fetal ventriculomegaly due to familial submicroscopic terminal 6q deletions. Prenat. Diagn..

[18] Eash D., Waggoner D., Chung J., Stevenson D., Martin C.L. (2005). Calibration of 6q subtelomere deletions to define genotype/phenotype correlations. Clin. Genet..

[19] Santen G.W., Aten E., Silfhout A.T.V.-V., Pottinger C., van Bon B.W., van Minderhout I.J., Snowdowne R., van der Lans C.A., Boogaard M., Linssen M.M. (2013). Coffin-Siris syndrome and the BAF complex: Genotype-phenotype study in 63 patients. Hum. Mutat..

[20] Stevens C.A., Fineman R.M., Breg W.R., Silken A.B. (1988). Report of two cases of distal deletion of the long arm of chromosome 6. Am. J. Med. Genet..

[21] Meng J., Fujita H., Nagahara N., Kashiwai A., Yoshioka Y., Funato M. (1992). Two patients with chromosome 6q terminal deletions with breakpoints at q24.3 and q25.3. Am. J. Med. Genet..

[22] Paulraj P., Palumbos J.C., Openshaw A., Carey J.C., Toydemir R.M. (2018). Multiple Congenital Anomalies and Global Developmental Delay in a Patient with Interstitial 6q25.2q26 Deletion: A Diagnostic Odyssey. Cytogenet. Genome Res..

[23] Shen-Schwarz S., Hill L.M., Surti U., Marchese S. (1989). Deletion of terminal portion of 6q: Report of a case with unusual malformations. Am. J. Med. Genet..

[24] Krassikoff N., Sekhon G.S. (1990). Terminal deletion of 6q and Fryns syndrome: A microdeletion/syndrome pair?. Am. J. Med. Genet..

[25] Valtat C., Galliano D., Mettey R., Toutain A., Moraine C. (1992). Monosomy 6q: Report on four new cases. Clin. Genet..

[26] Hecht F., Hecht B.K. (1992). Nonrandom chromosome breakpoints in 6q deletions. Clin. Genet..

[27] Denison S.R., Callahan G., Becker N.A., Phillips L.A., Smith D.I. (2003). Characterization of FRA6E and its potential role in autosomal recessive juvenile parkinsonism and ovarian cancer. Genes Chromosomes Cancer.

[28] Bartoshesky L., Lewis M.B., Pashayan H.M. (1978). Developmental abnormalities associated with long arm deletion of chromosome No. 6. Clin. Genet..

[29] Goldberg R., Fish B., Ship A., Shprintzen R.J. (1980). Deletion of a portion of the long arm of chromosome 6. Am. J. Med. Genet..

[30] Striano P., Malacarne M., Cavani S., Pierluigi M., Rinaldi R., Cavaliere M.L., Rinaldi M.M., De Bernardo C., Coppola A., Pintaudi M. (2006). Clinical phenotype and molecular characterization of 6q terminal deletion syndrome: Five new cases. Am. J. Med. Genet. A.

[31] Elia M., Striano P., Fichera M., Gaggero R., Castiglia L., Galesi O., Malacarne M., Pierluigi M., Amato C., Musumeci S.A. (2006). 6q terminal deletion syndrome associated with a distinctive EEG and clinical pattern: A report of five cases. Epilepsia.

[32] Nagamani S.C.S., Erez A., Eng C., Ou Z., Chinault C., Workman L., Coldwell J., Stankiewicz P., Patel A., Lupski J.R. (2009). Interstitial deletion of 6q25.2–q25.3: A novel microdeletion syndrome associated with microcephaly, developmental delay, dysmorphic features and hearing loss. Eur. J. Hum. Genet..

[33] Nair S., Varghese R., Hashim S., Scariah P. (2012). Dysmorphic features and congenital heart disease in chromosome 6q deletion: A short report. Indian J. Hum. Genet..

